# Current knowledges in pharmaconutrition: “*Ketogenics*” in pediatric gliomas

**DOI:** 10.3389/fnut.2023.1222908

**Published:** 2023-08-08

**Authors:** Nicola Cecchi, Roberta Romanelli, Flavia Ricevuti, Marianna Amitrano, Maria Grazia Carbone, Michele Dinardo, Ernesto Burgio

**Affiliations:** ^1^Clinical Nutrition Unit – A.O.R.N. Santobono-Pausilipon Children’s Hospital, Naples, Italy; ^2^Department of Translational Medical Science, Section of Pediatrics, University of Naples “Federico II”, Naples, Italy; ^3^ECERI-European Cancer and Environment Research Institute, Brussels, Belgium

**Keywords:** brain cancer, ketogenic diet, pediatrics, glioma, epigenetic

## Abstract

Brain tumors account for 20–25% of pediatric cancers. The most frequent type of brain tumor is Glioma from grade I to grade IV according to the rate of malignancy. Current treatments for gliomas use chemotherapy, radiotherapy, tyrosine kinase inhibitors, monoclonal antibodies and surgery, but each of the treatment strategies has several serious side effects. Therefore, to improve treatment efficacy, it is necessary to tailor therapies to patient and tumor characteristics, using appropriate molecular targets. An increasingly popular strategy is pharmaconutrition, which combines a tailored pharmacological treatment with a diet designed to synergize the effects of drugs. In this review we deal in the molecular mechanisms, the epigenetic effects and modulation of the oxidative stress pathway of ketogenic diets, that underlie its possible role, in the treatment of infantile gliomas, as a complementary approach to conventional cancer therapy.

## 1. Introduction

Pediatric gliomas represent the most common brain tumor in children. Histological grade is the most significant classification system affecting treatment planning and prognosis; based on histological criteria, the World Health Organization (WHO) introduced a malignancy classification system grading from I to IV (1). The incidence of brain tumors in children is about 5 cases per 100,000 population, 75% of which are classified as gliomas (2). 20% of pediatric gliomas are high grade gliomas (HGGs) and include anaplastic astrocytoma (AA), diffuse intrinsic pontine glioma (DIPG), and glioblastoma multiforme (GBM). WHO grades I–IV have extremely different 5-year survival rates up from 95% for grades I and II gliomas to 15–35% for grade IV gliomas or even less than 1% for DIPG (3). In recent years, thanks to the study of genetic variants, significant information associated with the prognosis and clinical course of glioma has been acquired. Clinical trials to date have benefited only limited subsets of patients, accentuating the fact that pediatric high-grade gliomas (HGGs) constitute an extremely heterogeneous group of highly aggressive brain tumors. DIPG is the most frequent brainstem tumor in the pediatric populations, and accounts for up to 80% of all pediatric brainstem tumors. DIPG is known to have a poor prognosis, with a median survival of about 11 months using current standard of care (4). Faced with this upsetting outcome, physicians often try to supplement the standard treatment with additional and alternative strategies, including complementary therapies. Glioblastomas are intrinsic brain tumors believed to originate from neuroglial stem or progenitor cells. Today’s dominant paradigm in the field of carcinogenesis can be described as stochastic mutational, a model where cancer is ultimately viewed as the product of a slow accumulation of stochastic DNA mutations. The fact that cancer increases especially in the early years of life is something unexpected and difficult to explain with the model of slow accumulation of stochastic mutations. For childhood neoplasms, it is safe to assume that cancer is the product of degeneration in a neoplastic sense of tissues undergoing very rapid proliferation and differentiation, in which proliferative and differentiative programs are being disturbed by increasingly early (maternal-fetal) exposure to a growing number of environmental stressors and pollutants.

The use of alternative and adjuvant therapies in pediatric cancer patients appears to be a frequent choice as reported in the reviewed literature. Surgery to the extent that this is feasible in terms of safety, followed by involved-field radiation therapy and concomitant and maintenance chemotherapy with temozolomide, has set the standard of care since 2005. Unlike most other cancers, where metastases from the site of origin is the main cause of death, GBM very rarely metastasizes outside the neuraxis. In this case, local invasion/recurrence of the tumor is the main cause of death (5). Infiltrative growth of malignant cells invading sane brain parenchyma, is a characteristic of GBM (6). One of the long-proposed possible mechanisms of resistance to treatment is cancer stemness, also reputed to be responsible for the ability of subpopulations to repopulate new metastatic niches (7). These cells exhibit properties of normal neural stem cells (NSCs), such as self-renewal and the ability to differentiate into definite progenies (8). Being somewhat resistant to chemotherapy and radiation therapy, if not completely removed during surgical resection, they have the ability to generate neoplastic (more aggressive) recurrences (9). Cancer and particularly cancer stem cells (which are the real problem, at the origin, for metastasis and for treatment resistance) use several complementary and/or alternative molecular pathways and mechanisms. Isolation of a subpopulation of glioma stem cells (GSCs) could help to understand the mechanisms and adaptation at the origin of invasiveness, for example, of GBM (10). Thus, the targeted therapeutic strategy is not necessarily the most effective and tends to select the stem cells not targeted by drugs that can only be derived from surgery.

## 2. KD represents an encouraging opportunity to target metabolic alterations in cancer cells

In recent years, there has been growing scientific interest in the use of the ketogenic diets (KDs) as a adjuvant approach to conventional cancer therapy, particularly against CNS tumors (11). KD represents an encouraging option to target metabolic alterations in cancer cells. Recent research shows that KD potentially acts as a tumor growth-limiting factor, protects healthy cells from chemotherapy or radiation damage, increases the toxicity of chemotherapy drugs on cancer cells, and reduces inflammation (12). KD is a high-fat, low-carbohydrate diet that has been used for decades as a non-pharmacological approach to treating neurological and metabolic disorders. The metabolic particularities of cancer, such as impaired mitochondrial activity, have led to an increase research on nutritional strategies that can successfully modulate the tumor responsivity during treatment.

### 2.1. History: from fasting to ketogenic diet

The ketogenic diet is a restrictive therapeutic diet with high-fat concentration, normoproteic, and hypoglucidic, and is considered a pharmaconutritional therapy (13). Since the 1920s, the ketogenic diet has been used successfully to treat patients with intractable epilepsy.

The term “ketogenic diet” was coined in 1921 by Dr. Russell Morse Wilder, of the Mayo Clinic, to describe a diet that produced a high level of ketone bodies, three water-soluble compounds, β-hydroxybutyrate, acetoacetate, and acetone, produced by the liver in otherwise healthy people when they were starved or on a very low-carbohydrate, high-fat diet (14, 15). The advent of phenytoin and other modern pharmaceutical anticonvulsants in the 1940s, and the subsequent lack of publications by epilepsy centers, meant that KD confined as an “alternative” medicine was largely ignored by mainstream neurologists. For many decades it was used only as a last recourse in children with intractable epilepsy, until 1993, when a refractory case sparked renewed interest in ketogenic diets. Hollywood producer Jim Abrahams took his 2-year-old son Charlie to Johns Hopkins Hospital, where Charlie started a KD and achieved a seizure control after few days. Abrahams created the Charlie Foundation for Ketogenic Therapies to further promote the dietary therapy (16).

Although glycemic modulation by carbohydrate-restricted diets is currently object of study in cancer research, clinical data are still lacking. Preclinical studies and early available clinical trials suggest positive effects on survival in patients with malignant gliomas (17). First studies of glucose utilization in cancer go back to the 1980s (18). The first case report published in 1995, illustrated the treatment of two female pediatric patients, with advanced stage malignant brain tumors (19), with KD based on MCT oil. They demonstrated that KD decreased the availability of glucose to the tumor without alter overall nutritional status. In summary, KD could have several beneficial effects in cancer treatment, but further supporting clinical data are needed (20).

### 2.2. “Ketogenics”

**Figure 1:**
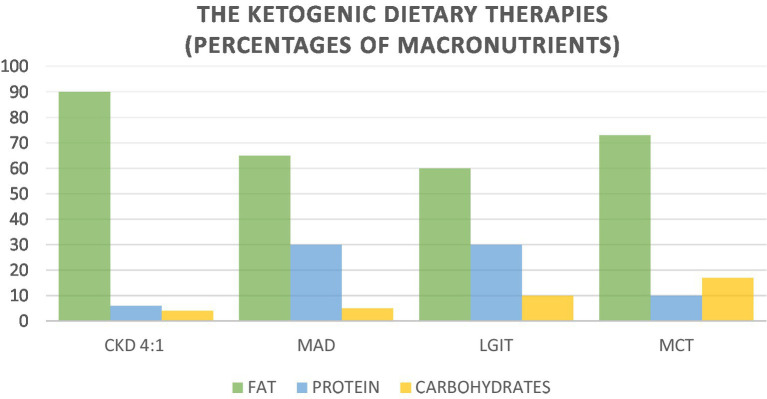
Nutritional characteristics of ketogenic dietary therapies.

In addition to the Classic Ketogenic Diet Protocol, alternative protocols have been released with the aim of increasing the percentage of carbohydrates to improve the patient’s compliance. These alternative protocols are proposed in a targeted manner as an adjuvant therapy to anticancer therapy. The ketogenic dietary therapies (KDTs) are the Classic Ketogenic Diet (CKD), the Ketogenic Diet with Medium-Chain Triglycerides (MCT), the Modified Atkins Diet (MAD), and the Low Glycemic Index Treatment (LGIT) (Figure 1).

The expectation of these diets is to induce a state of systemic ketosis.

The Modified Atkins Diet (MAD), involves a high lipid intake and significant carbohydrate restriction, while there is no specific range for proteins. Carbohydrates are limited to 10 g/day in the induction phase, and increased to 20–30 g/day after 1 month of treatment. Carbohydrates can be distributed between meals or included in a single meal. Dietary fiber is not calculated, and can be included in the dietary plan. Unlike the classical protocol, which involves precise calculation of macronutrients and calories, MAD allows greater freedom of food choice for the patient, making the diet very palatable and varied (21).

The Low Glycemic Index Treatment (LGIT) was developed in 2002 as an alternative to the CKD protocol. LGIT involves a daily carbohydrate intake equal to 10% of requirements, which corresponds to about 40–60 g/day, 60% of fat, and a protein intake according to individual needs. The LGIT protocol mandates that the glycemic index of foods should not exceed 50. This dietary approach produces comparable efficacy to the classical KD, but tolerability is Pfeifer & Thiele (22).

The ketogenic diet with Medium-Chain Triglycerides (MCT) was set up in 1971 by Dr. Huttenlocher and colleagues to replace the LCTs of CDK. The use of MCTs resulted in increased levels of ketosis, while maintaining good percentages of carbohydrates and proteins. The ketogenic diet with MCTs consists of 70–75% lipids, of which 60% is MCTs. The amount of MCTs may contribute to gastrointestinal disorders, including diarrhea, vomiting, bloating and abdominal cramps. For this reason, a gradual introduction of MCTs is recommended, starting at 30% of the total (23).

### 2.3. The biochemistry of the ketogenic diet

**Figure 2:**
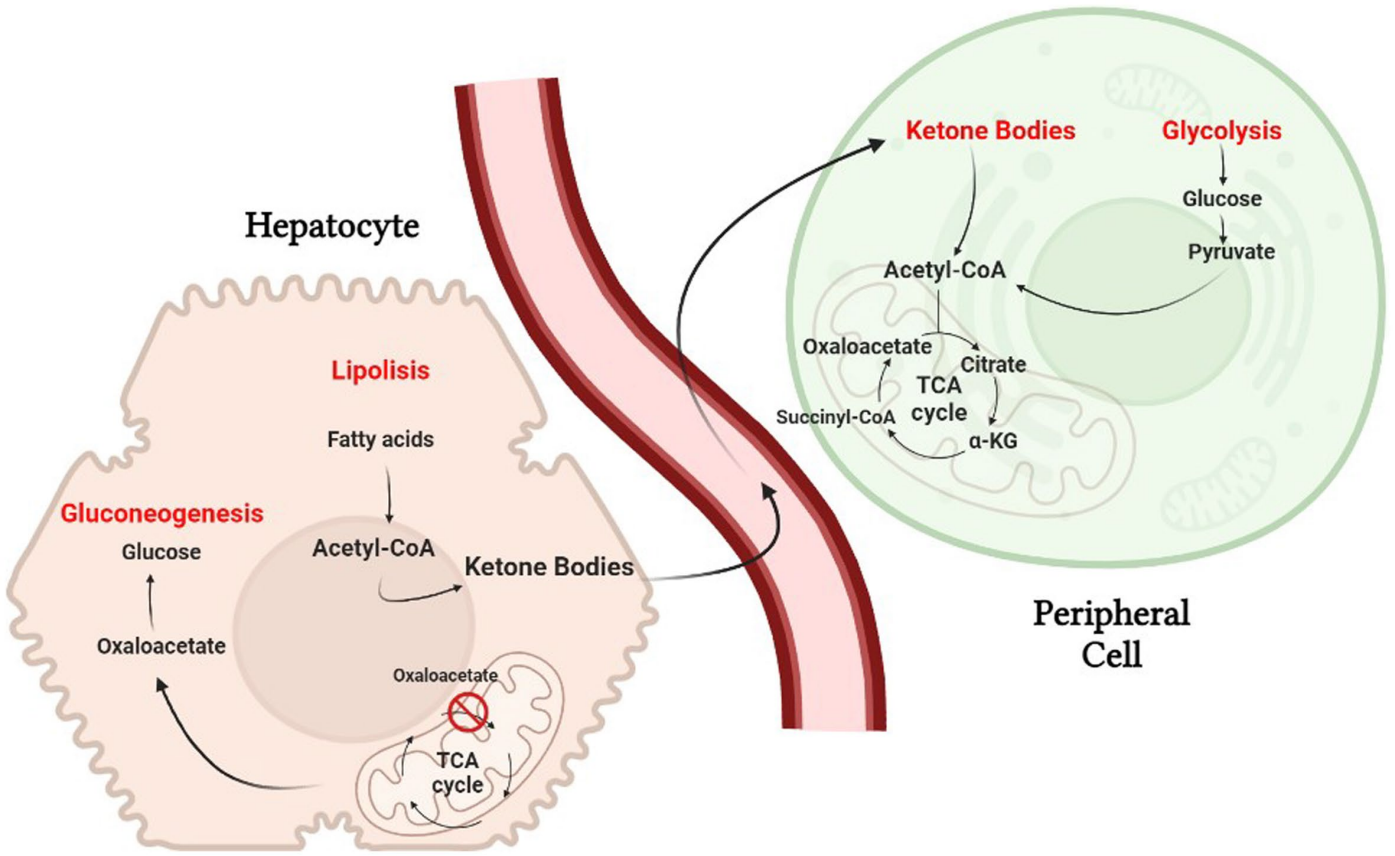
The biochemistry of the ketogenic diet. Hepatocytes must use all available oxalate for gluconeogenesis, causing a reduction of the TCA cycle.

The ketogenic diet emulate the body’s response to starvation or fasting by reducing carbohydrates. Liver hepatocytes convert fat from the diet into fatty acids and ketone bodies, allowing them to be used as alternative sources of energy. Ketone bodies, such as acetone, acetoacetate and β-hydroxybutyrate, cross the blood–brain barrier and are used as an alternative energy source to glucose (13). Because of the constant maintenance of blood sugar levels to avoid hypoglycemic coma, hepatocytes must use all available oxalate for gluconeogenesis, causing a reduction of the TCA cycle; nevertheless, acetyl-CoA will continue to be abundantly produced through beta-oxidation of fatty acids, causing its cytoplasmic accumulation. The excess acetyl-CoA is then used for the production of acetoacetate, which will be spontaneously converted to acetone (a volatile compound that is eliminated with exhalation) and 3-β-hydroxybutyrate, the predominant ketone body, by 3-β-hydroxybutyrate dehydrogenase (24). Once produced, the ketone bodies are released into the bloodstream and reach peripheral cells where they are converted back to acetyl-CoA. Peripheral tissues, that are unable to do gluconeogenesis and have availability of oxaloacetate, use acetyl-CoA in the Krebs cycle (Figure 2).

## 3. Ketogenic effect on neoplastic cells

**Figure 3:**
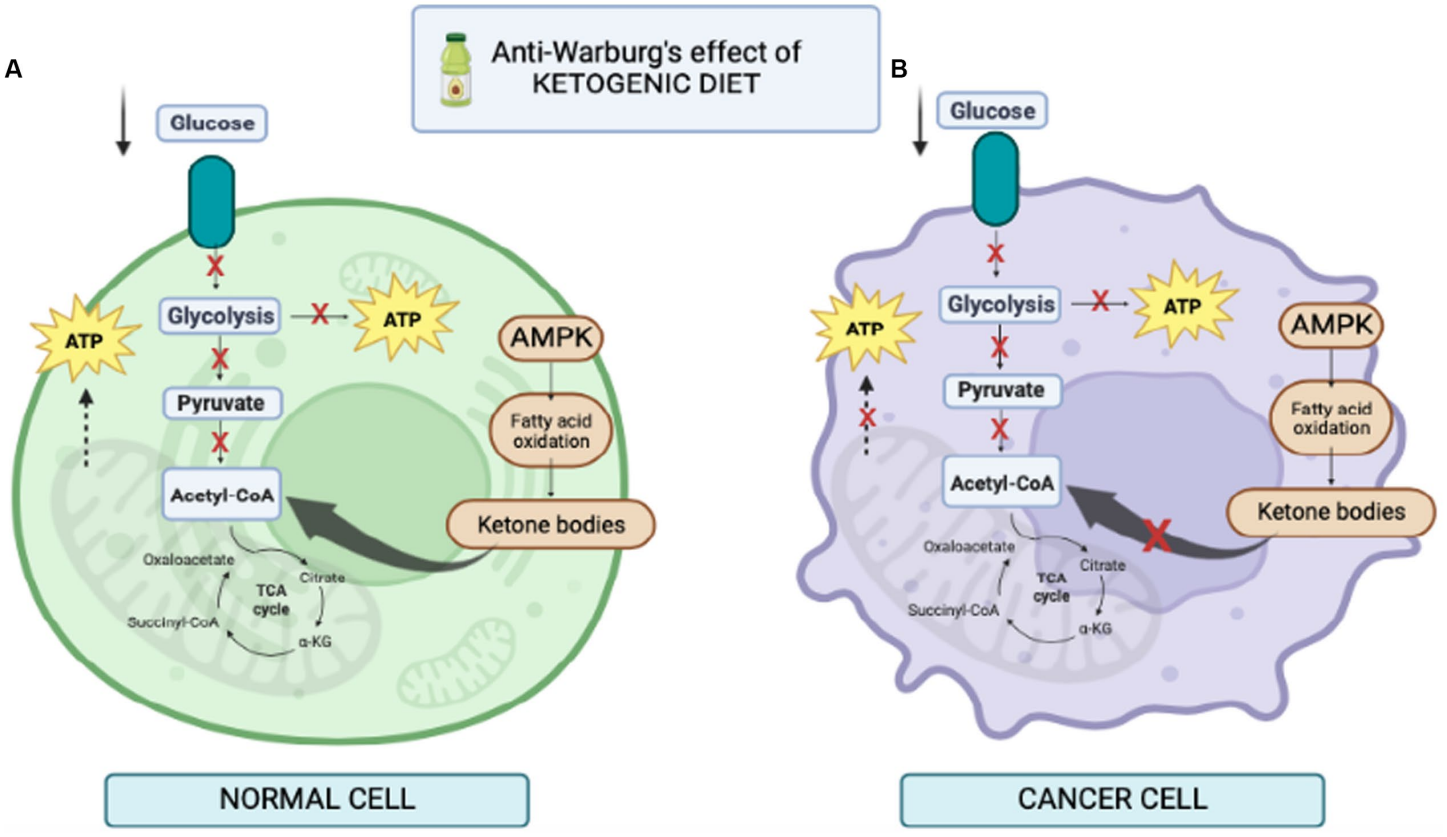
Illustration of cell behavior during ketogenic diet. **(A)** in normal cells fed KD, lower level of glucose increases the level of ketone bodies as a result of the increase in free fatty acids, thus increasing the level of acetyl-CoA in mitochondria for production of ATP. **(B)** in cancer cells fed KD, it reduces glycolysis. In addition, mitochondria may be dysfunctional and the cells are unable to produce ATP; therefore, KD prevents cancer cell proliferation.

Normal brain cells and cancer cells may differ in their ability to utilize ketone bodies as metabolic fuel (Figure 3). Under physiological conditions, normal brain cells can obtain energy from both glucose or ketones (25). On the other hand, some investigations demonstrated that many tumors have reduced expression of crucial ketolytic enzymes. Notably, glioma cells showed an inability to metabolize ketone bodies *in vitro*, suggesting a potential disadvantage of tumor cells compared to normal cells under a low-carb ketogenic diet (26, 27).

Additionally, KD could be able to modulate gene expression both directly and indirectly through epigenetic modifications. Many studies have shown a neuroprotective role of ketogenic diet by reducing the production of reactive oxygen species. The identification of β-hydroxybutyrate as an NLRP3 inhibitor encourages further studies on the efficacy of a ketogenic diet on the inflammatory mechanisms of cancer cells.

### 3.1. The Warburg effect

Warburg and his co-workers demonstrated in the 1920s that, cultured tumor tissues, despite the availability of oxygen, uptake high amounts of glucose metabolized to lactate, a phenomenon known as aerobic glycolysis. These metabolic properties constitute the Warburg effect. Long before Warburg, Pasteur demonstrated that sugar fermentation is suppressed in the presence of oxygen, thus identifying the conversion of glucose to lactate as an expected response to hypoxia (28). However, cancer cells, before exposure to hypoxic conditions, appear to use glycolytic metabolism (29). Warburg thought aerobic glycolysis was a symptom of impaired oxidative metabolism, believing cancer to be a mitochondrial metabolic disease (30). Despite the extensive scientific evidences describing mitochondrial anomalies in various cancers, many investigators have demonstrate that mitochondria and OXPHOS are not seriously compromised or even intact in some cancer cells (31). In fact, also Warburg’s own experiments revealed persistent oxygen consumption in tumor tissue. Besides OXPHOS, approximately 11% (4/36 total ATP molecules) of total cellular energy is produced through substrate-level phosphorylation (SLP) reactions in the mitochondria (2 ATP) and cytoplasm (2 ATP). For this reason, the mol of ATP synthesized per mol of oxygen used (P/O ratio) is not showing the mitochondrial function in particular in cancer cells (32). One possibility is that the Warburg effect allows cells to maintain large pools of glycolytic intermediates that promote utilization of the pentose-phosphate pathway and other biosynthetic pathways branching off from glycolysis. Respiration promote cell proliferation by allowing the production of tricarboxylic acid (TCA) cycle intermediates necessary for anabolism. Mitochondrial DNA sequencing of 1,675 tumor biopsies from over 30 different types of cancer revealed a number of mutations that have deleterious effects on respiration (33). Other evidence of mitochondrial activity in human tumors revealed significant fuel oxidation in human brain and lung tumors (34, 35). Impaired oxidative metabolism may stimulate aerobic glycolysis in cancer, but, conversely, aerobic glycolysis does not predict loss of oxidative metabolism. This crucial conceptual framework of bioenergetic balance suggests that cancer cells are as dependent on the Warburg effect as are undifferentiated cells, the stem cells and multipotent cells, that can obtain mainly ATP from glycolysis rather than from OXPHOS, even in the presence of sufficient oxygen (29). This choice in metabolic control thus results from a return to embryonic/stem epigenetic programming and the ability to program cellular metabolism in a way that sustains cell growth, activating proliferative versus differentiative programs (36). A better understanding of the mechanical links between cell metabolism and growth control may ultimately lead to improvements in current cancer treatments. Pharmaconutrition, e.g., ketogenic diets, should be used early, especially immediately after surgery to reduce the glucose inflow and prevent triggering the Warburg effect.

### 3.2. The epigenetic effect

Epigenetic modifications are biochemical alterations in DNA or chromatin structure that do not alter the sequence of DNA, but affect the regulation of gene transcription. The epigenome can thus be defined as the organism’s ability to respond and adapt to environmental changes (37). Nutritional assessment and his factors, metabolites, nutrients, demonstrate to be a promising and important epigenetic modulators; growing scientific literature suggests that epigenetic changes have a connection with the ketogenic diet (38). The effect of KD on the genome and gene expression is still largely to be investigated. KD might be able to modulate gene expression directly or indirectly through various mechanisms such as the regulation of DNA methylation, modification of histone conformation, by acetylation, methylation, phosphorylation, ubiquitylation and beta-hydroxybutyrylation of lysine. Beta-hydroxybutyrylation of histones would seem to be exclusive to ketone bodies. These epigenetic modifications could underlie the ability of KD to modify the expression of oncogenes and tumor suppressors (39). Indeed, it has been observed that DNA methylation, miRNAs, and histone modifications occur during the early stages of cancer progression and metastasis (40). In glioblastoma (GBM), epigenetic modifications could modulate the inflammatory gene-oncogene loop and associated immunosuppression (41), modifying the inflammatory tumor microenvironment and playing a key role in the mechanisms of carcinogenesis. Increasingly accurate knowledge of these mechanisms could pave the way for new therapeutic strategies targeting the inflammatory tumor microenvironment.

#### 3.2.1. DNA methylation

The process of DNA methylation is mediated by enzymes belonging to the DNA methyltransferase (DNMTs) family (42). The main methyl-donor, S-adenosylmethionine (SAM), transfer a methyl group to the C5 position of cytosine within CpG dinucleotides and the formation of 5-methylcytosine (43).

**Figure 4:**
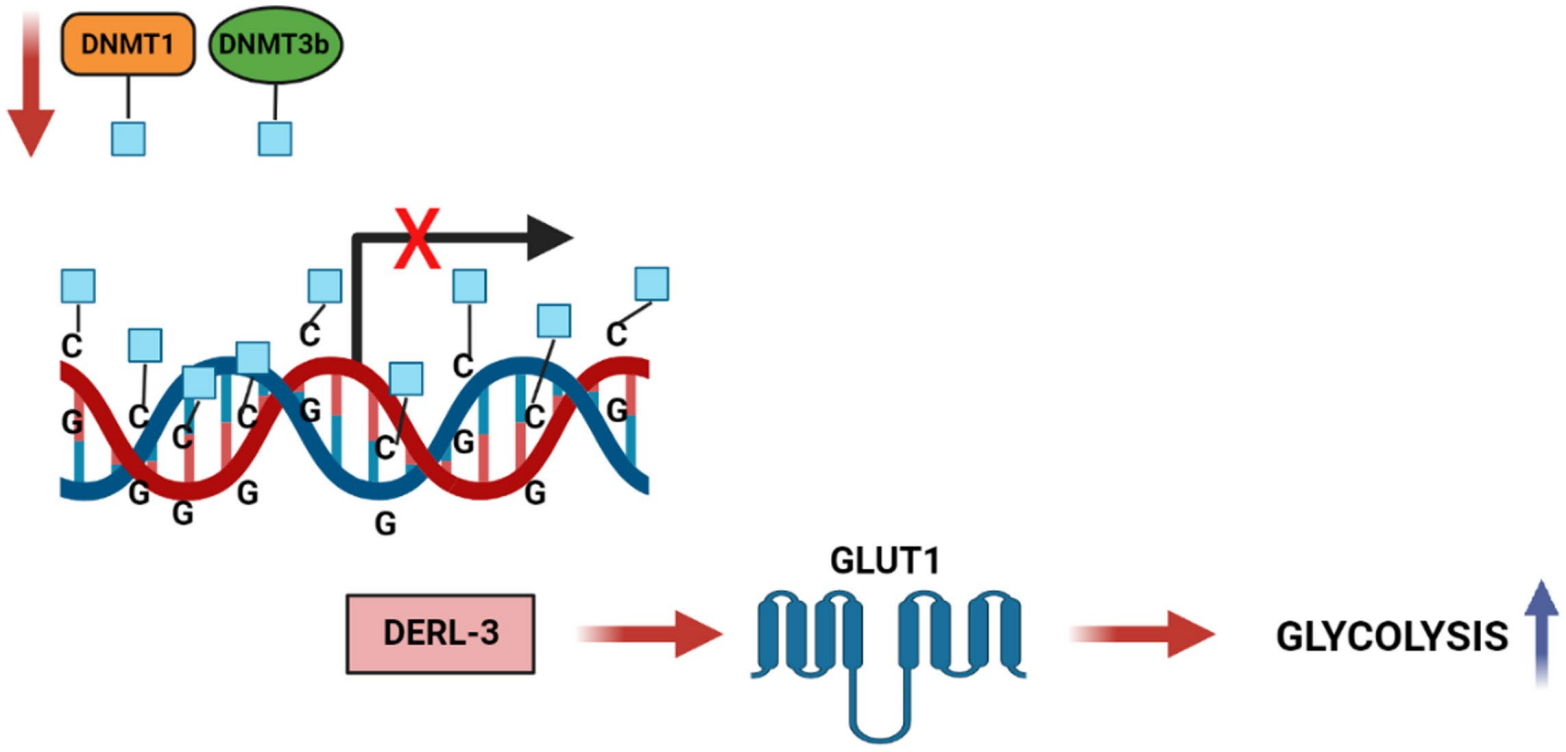
DNA methylation regulates Carbohydrate metabolic pathways in cancer cells. DNA hypermethylation of the DERL3 promoter by DNMT1 and DNMT3b lead to the upregulation of GLUT1 in cancer cells. This drives to an increased glucose uptake and a high rate of glycolysis in tumor cells.

Ungaro et al. in 2022 showed the effects on a rat model with chronic epilepsy of KD on DNA methylation (44). KD induces the increase in adenosine in the hippocampus (45) that is known to promote the formation of S-adenosylhomocysteine (SAH) (46), which blocks DNA methyltransferase (47), reducing global DNA methylation (46). The conversion of SAM to S-adenosyl-homocysteine (SAH) inhibits DNMTs and histone methyltransferases HMTs, thus dictating their enzymatic activities (48). The SAM/SAH ratio, regulated by glycine N-methyltransferase (GNMT) and referred to as the “methylation index,” is crucial in transcriptional regulation by chromatin modification. There is ample evidence that DNA methylation has an impact on cancer-related metabolic pathways. The epigenetic process of DNA methylation can modulate, directly or indirectly, gene expression of metabolic enzymes resulting in reprogramming of cancer cells. Direct modulation involves alteration of transcription by methylation or demethylation at the promoters of genes coding for proteins implicated in the metabolic pathway, while indirect methods include modification of oncogenic pathways, such as mTOR, Akt, HIF1 etc., acting downstream on specific metabolic intermediates (49). An example of indirect regulation (Figure 4) (50) of glycolytic genes in cancer is the hypermethylation of the Derlin-3 promoter, resulting in the increased expression of glucose transporter 1 (GLUT1). This significantly increases the uptake of glucose and its catalysis into final metabolites – lactate and pyruvate – which leads to acidification of the microenvironment, promoting tumor proliferation and invasion. One more impact of DNA methylation on the metabolism of cancer cells is the silencing of tumor suppressor genes, including PTEN (51), VHL (52), and PHD (53), that regulate various signaling pathways.

**Figure 5:**
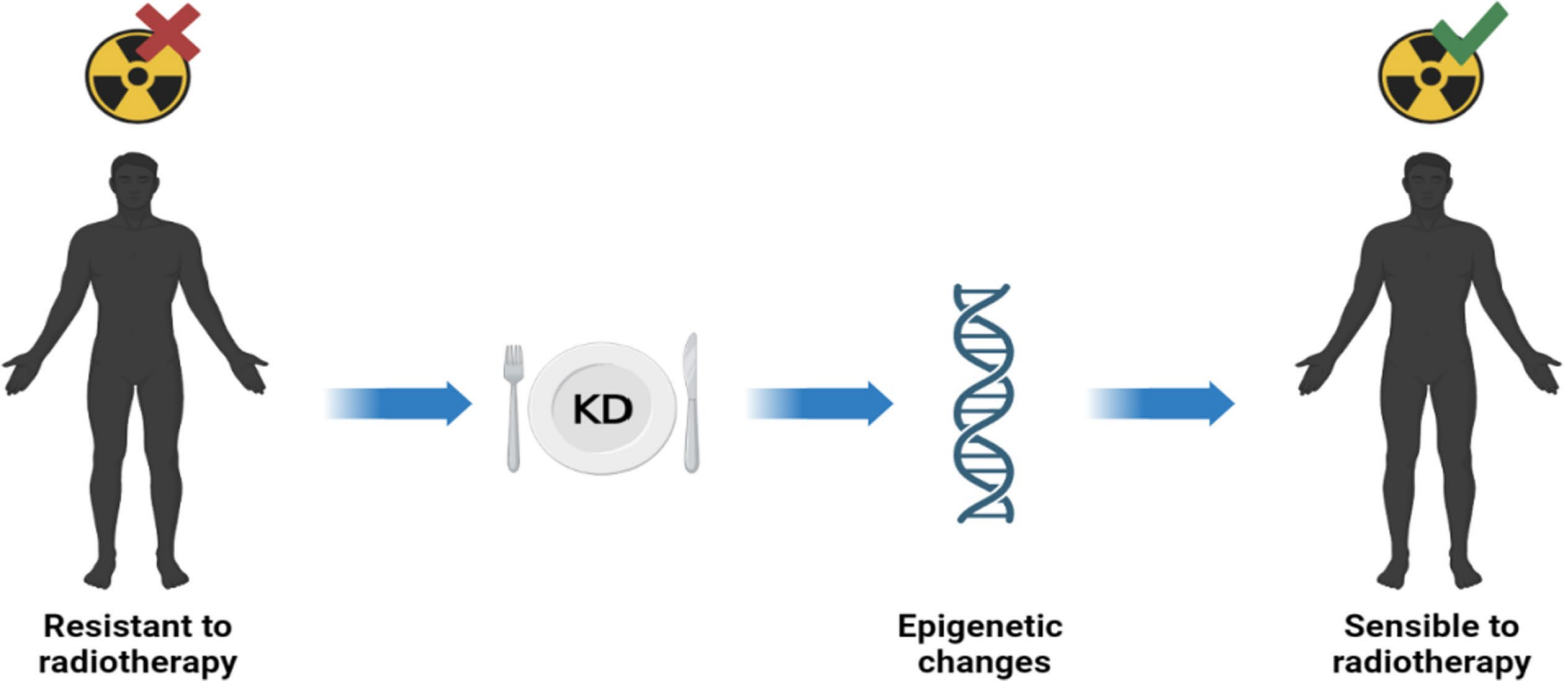
Ketotherapy and epigenetic modifications. KDs could act as epigenetic drugs by sensitizing radiotherapy-resistant patients. Radiosensitivity would be promoted by some epigenetic modifications by KDs (40).

Previous studies have reported that conventional therapies such as radiotherapy and chemotherapy can act on DNA methylation and inducing epigenetic reprogramming of cells. However, there is a link between DNA methylation and treatment response: chemo and radio therapies affects DNA methylation, but concurrently DNA methylation affects the response to treatments (54). Radiotherapy is well known as the most cost-effective treatment for many cancers in adults and children. However, some cancers exhibit an intrinsic resistance to radiation compared with healthy tissues; this feature is related, at least in part, to the tumor’s epigenetic characteristics. There are drugs that can sensitize tumors to treatment. The best-known epigenetic inhibitor drugs are DNMT1 inhibitors, which sensitize many tumor types to radiation (55). Ketotherapy is an epigenetic modifier, so KDs could as epigenetic factor, or as a complementary approach with anticancer drugs, or a synergistic action (Figure 5).

#### 3.2.2. Histone modifications

Histone protein modifications affect most processes involving DNA, including DNA packaging and chromatin compaction, nucleosome dynamics, and transcription (56). Recently, ketone bodies have been shown to act as epigenetic modifiers resulting in covalent modifications at key histones (39, 57). These include lysine acetylation, methylation, and lysine β-hydroxybutyrylation (Kbhb) (58). Inibition of class I histone deacetylases (HDACs) by β-Hydroxybutyrate has been demonstrated *in vivo* and *in vitro* (59); the HDACs are enzymes that works by removing acetyl from lysine residues on histone and non histone proteins and regulating gene expression. Class I HDACs (e.g., HDAC1, HDAC2, HDAC3, and HDAC8) are primarily located in the nucleus and are usually composed in large multiprotein regulatory group. Histone hyperacetylation, induced by histone acetyl transferases (HATs) or by inibitors of class I HDACs, is associated with chromatin remodeling and in the regulation of gene transcription (60). Β-OHB causes hyperacetylation of histones at the FOXO3a promoter, resulting in increased FOXO3a expression (61). FOXO3a is a transcription factors that regulate a variety of cellular processes including apoptosis (62), proliferation (63), cell cycle progression (64), DNA damage (65) and tumorigenesis. Indeed, overexpression of FOXO3a has been demonstrated to inhibit tumorigenesis (66) and also suppresses oxidative stress by inducing the expression of antioxidant factors such as metallothionein 2 (Mt2), manganese superoxide dismutase (MnSOD) and catalase, with increased acetylation of lysine residues in histone H3 via HDAC inhibition (67). It has been shown that in fasting condition, comparable with Classical Ketogenic Diet is able to increase overall levels of lysine acetylation of p53 improving its transcriptional activity. The authors hypothesized that p53 hyperacetylation may have an antiproliferative effect that contributed to the decrease in cancer incidence in mice fed KD (68). Some histone hyperacetylation following β-OHB treatment could be a consequence of the increase in the intracellular pool of acetyl-CoA (69). The wide efficacy of high-fat diets is also related to increased levels of the coenzyme nicotinamide adenine dinucleotide (NAD) (70), a pivotal molecule for redox reactions and essential of ATP generation. Since fewer NAD molecules are reduced during brain metabolism based on ketones than during glucose metabolism, high levels of the oxidized form (NAD^+^) can be expected. NAD^+^ serves as a substrate for two groups of enzymes, sirtuins and poly (ADP-ribose) polymerases (PARPs), which influence various cellular functions ranging from gene expression to DNA repair, stress responses and aging. For instance, the deacetylase SIRT3 acts as a tumor suppressor in several cancer cells and contributes in deacetylating PDHA1, a subunit of the pyruvate dehydrogenase complex (PDC), thereby inducing its activity and causing an anti-Warburg effect via the HIF1alpha/PDK1/PDHA1 pathway (71).

#### 3.2.3. β-Hydroxybutyrylation

**Figure 6:**
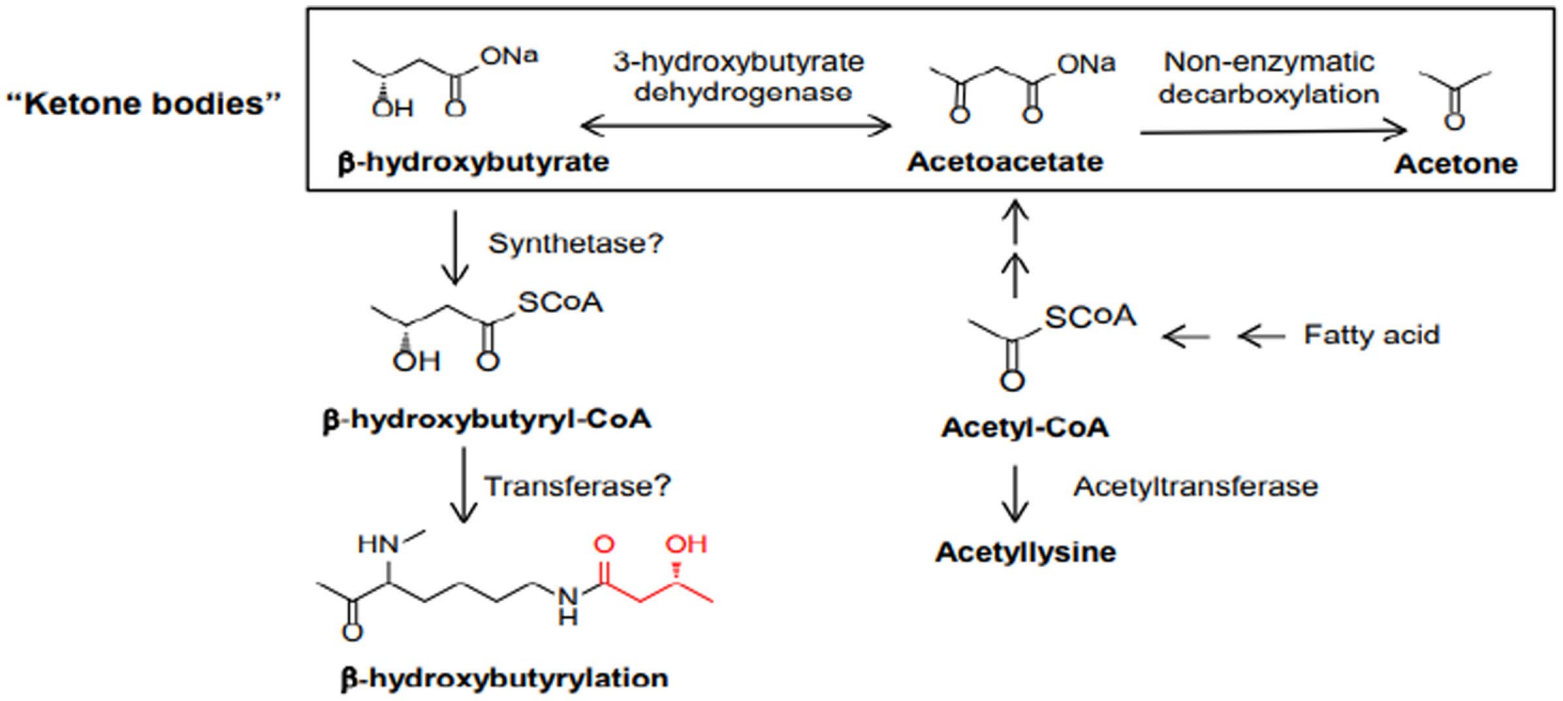
Biosynthetic pathways of b-hydroxybutyrate and b-hydroxybutyryl-CoA. Also depicted are the three ketone bodies: b-hydroxybutyrate, acetoacetate, and acetone.

Studies suggest that ketone bodies are involved in the coordination of cellular functions via a novel histone mark, β-hydroxybutyrylation (Kbhb) (72). As recently known Kbhb levels are significantly increased under conditions of fasting, high-fat diets, ketogenesis. β-OHB serves as a substrate for histone lysine b-hydroxybutyrylation (Kbhb) via its activated thioester form β-hydroxybutyryl-CoA (Figure 6). Since Kbhb sites overlap with lysine residues, this novel modification of histones complements the classical histone lysine acetylation, methylation, phosphorylation and ubiquitination, producing changes in chromatin status. In mice livers subjected to particular conditions, Xie et al. identified 44 lysines in histone proteins susceptible to β-hydroxybutyrylation, including H1K168, H2AK5/K125, H2BK20, H3K4/K9/K14/K23 and H4K8/K12 (73).

β-OHB serves as a substrate for histone lysine b-hydroxybutyrylation (Kbhb) via its activated thioester form β-hydroxybutyryl-CoA (Figure 6).

A huge increase in histone Kbhb occurred in a dose-dependent manner. In response to prolonged fasting or a ketogenic diet Quantitative Mass Spectrometry showed that the levels of most histone Kbhb sites increased by 10–40 fold (73). It also demonstrated that key enzymes in the metabolism of ketone bodies, such as 3-Hydroxy-3Methylglutaryl-CoA Synthase 2 (HMGCS2) and b-hydroxybutyrate dehydrogenase 1 (BDH1), affect the modification of histone Kbhb in the following way: loss of HMGCS2 in the intestine impairs H3K9bhb aggregation and affects H3K9bhb-related metabolic gene programs (74). As a consequence with the increase in histone Kbhb modification, the expression of several genes involved in physiological responses to fasting (or to high-fat diet) such as amino acid catabolism, redox balance, circadian rhythm, and PPAR (peroxisome proliferator-activated receptor) signaling was upregulated. These newly identified histone post-translational modifications represent novel hallmarks of epigenetic regulation that is able to linking metabolism to gene expression, suggesting a new approach to study chromatin regulation and the different functions of β-OHB in human pathophysiological states (73). Kbhb was initially identified on histones. However, potential substrates of non-histone proteins remained unknown. A study by He Huang et al. (75) the characterization of Kbhb is performed in HEK293 cells and provided the first global dataset of the Kbhb proteome, which contains 3,248 reliable Kbhb sites on 1,397 proteins. At the same time a study by Koronowski et al. (76) focused on AHCY, a rate-limiting enzyme that hydrolyzes S-adenosylhomocysteine (SAH) into homocysteine and adenosine. Western blots showed that both starvation and KD induced Kbhb on AHCY compared to control conditions. The results indicate that β-OHB (and probably Kbhb) inhibits AHCY activity, in conjunction with altered methionine cycle function. Cancer cells are highly dependent on methionine metabolism and several studies report AHCY as a possible therapeutic target, so global protein b-hydroxybutyrylation could play a significant role in the metabolic adaptation of cancer (77, 78).

#### 3.2.4. Modulation of microRNAs

MicroRNAs (miRNAs) are small non-coding RNAs of approximately 20–22 nucleotides (79). MicroRNAs are processed from stem-loop regions of longer RNA transcripts (80). They regulate gene expression by binding to target mRNAs to modulate their intracellular concentrations and to control the protein translation process (81). Genes for miRNAs are located in both in coding and non-coding regions (introns or exons), most of which are clustered (82). The pre-miRNA hairpin is transported in the cytoplasm by exportin-5 and a strand of the miRNA remains as a mature miRNA (guide strand) that recognizes the complementary sequence on the target mRNA (83). When miRNAs are upregulated they could act as oncogenes (oncomirs) silencing the oncosuppressor genes (84), conversely, downregulated miRNAs could suppress the tumor process (85). A systematic review of miRNAs in GBM identified 253 overexpressed and 95 underexpressed miRNAs, while 17 miRNAs are variously regulated (86). There are also several miRNAs that target oncogenes and play tumor suppressor roles in GBM. When there is a overexpression of these tumor suppressor miRNAs, there is an inhibition of tumorigenesis and tumor progression. The use of KD in mouse models of glioblastoma has shown a modulation of the expression of various miRNAs, resulting in a reduction in tumor progression and an increase in long-term survival (87). The impact of KD on reducing tumor development and improving survival of malignant glioma models in animals has been demonstrated in preclinical trials by modulating miRNAs (88, 89). KD increases the expression of several miRNAs, implicated in the progression of glioma (90). More investigations are important to determine the potential role of miRNA and the ketogenic diets in cancer development.

### 3.3. Modulation of oxidative stress

**Figure 7:**
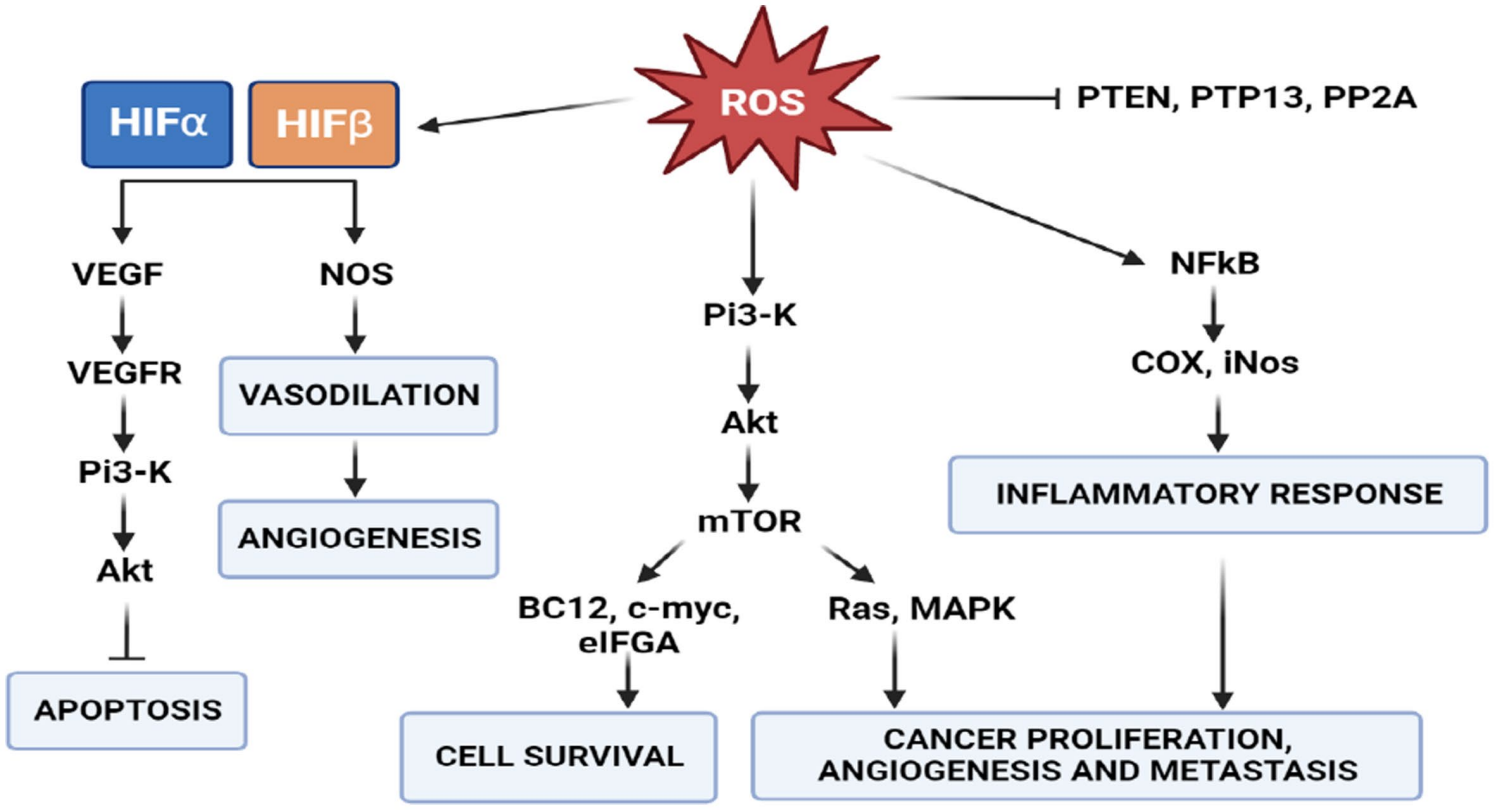
Role of ROS in signal transduction. PI3K/AKT/mTOR signaling activates two major signaling pathways: Ras-MAPK, which leads to cell proliferation, and PI3K-Akt-eNOS, determines cell survival. In cancer cells, a high concentration of ROS activates the survival pathway and inactivates the PTEN pathway to avoid apoptosis.

Many studies have established that one of the phenotypes of cancers is increased production of reactive oxygen species (ROS) and oxidative stress (91). ROS are highly reactive oxygen-containing molecules. They are produced in mitochondria, peroxisomes and the endoplasmic reticulum during various biochemical processes. ROS are also generated after exposure to physical agents (ultraviolet radiation and heat) and after chemotherapy and radiotherapy in cancer. Increased ROS levels constitute one of the many hallmarks of a cancer cell. These changes in cancer cells are characterized by respiratory dysfunction, low coupling efficiency of the mitochondrial electron transport chain, increased electron leakage and amplified ROS generation (92). These mechanisms underlie cancer development, including initiation, promotion and progression. Indeed, increased intracellular levels of ROS can result in the activation of oncogenes and oncogenic signals involved in apoptosis, angiogenesis, cell survival and cancer proliferation and metastasis (50) (Figure 7). However, this increase in oxidative stress is maintained at borderline levels by up-regulation of endogenous antioxidant systems (93), allowing them to survive and proliferate in a redox state unlike healthy cells.

**Figure 8:**
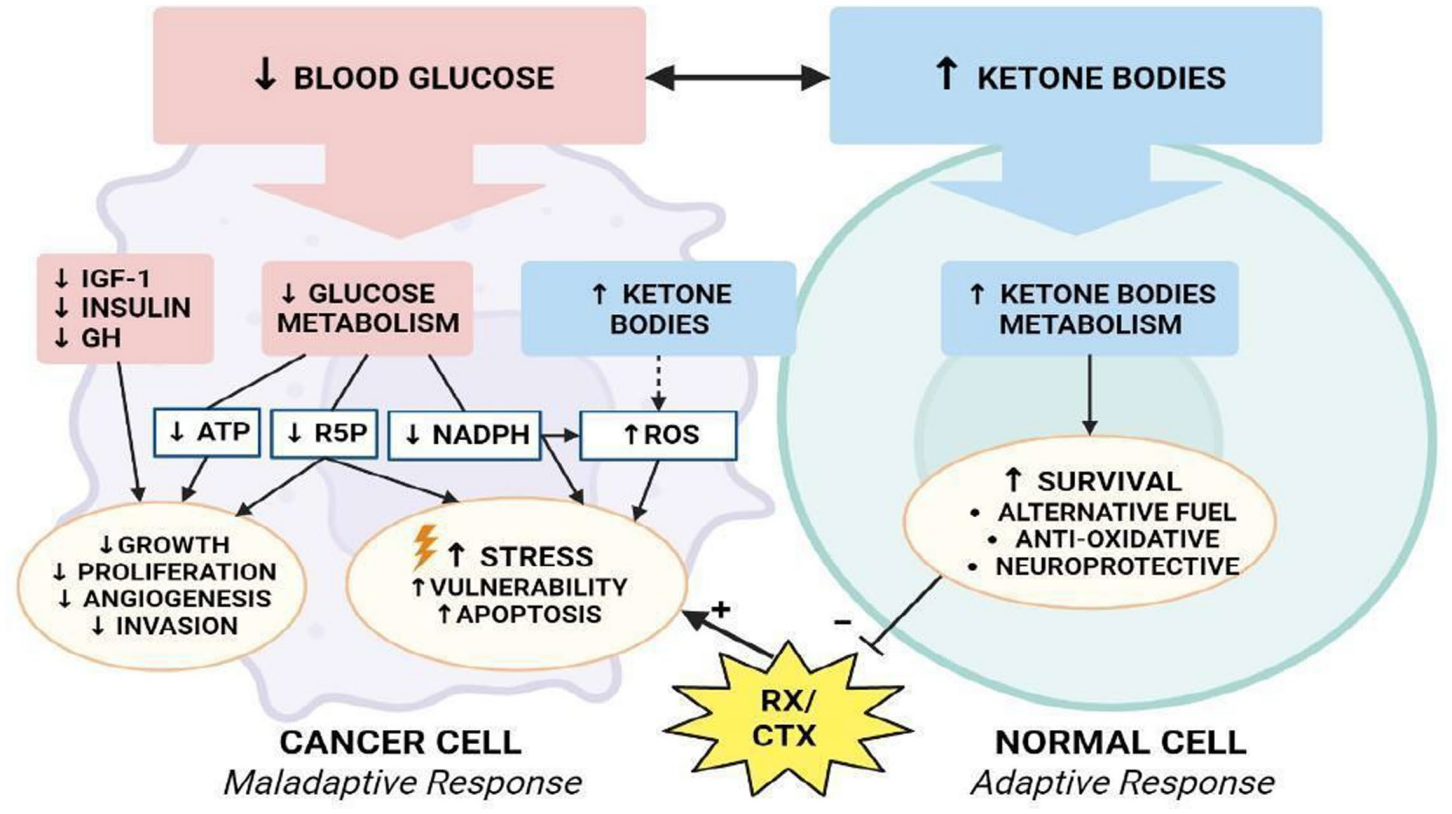
Modulation of oxidative stress. How KD differentially affects metabolism of normal and malignant brain cells.

There is, however, a threshold points beyond which ROS irreversibly damage and induce apoptosis in cancer cells. This phenomenon has stimulated the of pro-oxidant therapies, such as radiation and chemotherapy, with the intention of disrupting the oxidative balance to induce cell death (94). Many studies demonstrated that the ketogenic diet also elevates oxidative stress in tumor cells, while simultaneously being neuroprotective the production of reactive oxygen species via the mitochondrial Co-enzyme Q pair in normal cells (95). Several studies have shown that a state of ketosis protects healthy cells from oxidative stress by simultaneously decreasing the production of ROS and increasing that of antioxidants. In normal cells, KD may increase the expression of mitochondrial uncoupling proteins (UCPs) through the regulation of Sirtuin 1 activity (96), potentially by altering NAD^+^ levels, although the precise mechanisms are not yet known. Increased UCP activity reduces the mitochondrial membrane potential and lowers the production of ROS and reactive nitrogen species. In addition, BHB inhibits HDAC, leading to an increase in histone acetylation on the FOXO3a transcription factor promoter (97), which induces the expression of the antioxidant proteins superoxide dismutase (SOD) and catalase, resulting in a reduction of ROS. Conversely, intracellular accumulation and active KB signaling could further disrupt cancer cell homeostasis, by affecting ROS levels and glycemic stress-inducing reduction in metabolically maladaptive brain cancer cells (Figure 8) (98).

The reduction of glycolysis process (↓ATP), decreased redox potential (↓NADPH), and reduced availability of ribose-5-phosphate will lead cancer cells toward a an apoptotic process and confer vulnerability to cytotoxic chemo-and/or radiotherapy (99). In addition, a reduction in GH, IGF-1 and insulin levels further reduces the processes of proliferation, angiogenesis and invasion of tumor cells, reducing the pro-survival stimuli mediated by Akt/mTOR and Ras/MAPK signaling (100). In one study, researchers examined the effects of KD, radiation, and chemotherapy on oxidative stress in cancer cells (101). They demonstrated that animals fed KD in combination with radiotherapy showed an increase in lipid peroxidation and oxidative damage that could be related to a reduction in metabolic substrates with antioxidant action.

### 3.4. Inibition of inflammasome activation

Inflammasome activation is essential for the host to counteract external pathogens or tissue damage, while abnormal inflammasome activation can cause runaway responses that can contribute to autoinflammatory disorders, cardiometabolic diseases, cancer, and neurodegenerative diseases. When innate immune receptors are activated by various stimuli, transcription of pro-inflammatory genes is induced, including those encoding NLRP3 and pro-IL1β, but other pathways are now also known. The transcribed pro-inflammatory genes are components of the NLRP3 inflammasome complex. The NLRP3 inflammasome is involved in the molecular mechanism of many autoinflammatory diseases.

Inflammasomes can be activated by different stimuli, including microbiome-derived and host-derived signals. Upon activation, inflammasome sensors NAIP/NLRC4, NLRP3/6/7, AIM2/IHI16 initiate inflammasome assembly by recruiting pro-caspase-1 filaments. It is mandatory to maintain a fine balance between inflammasome activation and inhibition, which requires a refine of inflammasome assembly and effector function. What do we now know about the therapeutic role of the ketogenic diet in regulating the fine balance of the inflammasome?

The identification of β-hydroxybutyrate as an NLRP3 inhibitor provides a rationale for investigating the efficacy of an anti-inflammatory ketogenic diet for the treatment of NLRP3-dependent autoinflammatory diseases. Several mechanisms by which ketogenic inhibits or regulates the inflammasome have been identified in many papers, such as:

- BHB acts as an NLRP3 inflammasome inhibitor. Activation of HCAR2 by BHB leads to activation of the enzymes PGDS (Prostaglandin-D synthase) and COX1 (cyclooxygenase 1), which induce prostaglandin synthesis, reducing NF-κB activation, decreasing proinflammatory genes, and activating the transcription factor PPARgamma. In addition, ketogenic diet is associated with overexpression of ARRB2 (Beta Arrestin 2), which directly inhibits NLRP3 activity (102).
- BHB inhibits IL-2 production by mast cells in part through GPR109A signaling (103).
- BHB inhibits the secretion of IL-1β by neutrophils and IL-18 (104).
- Ketogenic diet and BHB inhibit NLRP3 activation in aged neutrophils
- BHB inhibits both the priming and assembly phases of NLRP3 activation in neutrophils.

### 3.5. Activity in tumor microenvironment

Rearrangement of tumor microenvironment (TME) components through mutual and dynamic crosstalk is an important factor influencing the process of tumor formation and progression (105). The composition of the TME differs among tumor types, but include cells of the immune system, a convolute network of stromal cells, fibroblasts, blood vessels, and extracellular matrix (106). GBM cells can manipulate the microenvironment around them by developing a niche that supports tumor growth and development (107), and in this tumor TME is is likely to be the key factor compromising treatment efficacy. Non-tumor glial cells and endothelial cells can directly interact with GBM cells and/or modulate GBM metabolism, release soluble molecules such as inflammatory factors, triggering signaling pathways that promote cell invasion, tumor progression and development (108). In one study, researchers demonstrated that a Classical Ketogenic Diet can potentially be used to alter the glycolytic phenotype of GBM’s cells, differently in a murine models the high-fat diet has been show to modify the polarization of macrophages toward a paradoxical “pro-tumor” immune-suppressive response. Mitigation of this immune response, the well known anti-inflammatory properties of this diet appear to be a consistent perspective themes of investigation. Mitochondrial dysfunction (Warburg effect) is not a general feature of all cancer cells within the tumor mass, as has been demonstrated by the existence of oxidative tumor cells. The numerous reports suggesting that OXPHOS is normal or not severely impaired in GBM cells is not consistent with this basic principle.

## 4. Literature review

A substantial number of preclinical and clinical studies have been performed to evaluate the effect of KD on low-grade and high-grade glioma. In clinical studies, many endpoints were evaluated: tumor growth, accumulation of ketone bodies in glioma cells, tolerability and adverse effects of the diet, survival rate. On children only two descriptive studies have been conducted, but RCTs and large-sample studies are lacking.

### 4.1. Preclinical studies

Several preclinical studies have shown that KD reduces tumor symptoms (oedema, hemorrhage) and tumor volume and prolongs the survival rate (Table 1). Thus, KD modulates gene expression by reducing ROS and oxidative stress molecules, blocks the energy metabolism of cancer cells and enhances cell death through apoptosis. Although KD alone does not achieve the same results, it enhances the effect of chemotherapy on tumor cells. The treatment is safe and feasible: only a few patients have reported mild side effects, such as borderline glycaemia and/or hyperketonemia. Stafford et al. showed that KD treatment reduces the tumor growth rate and prolongs survival. KD modifies the gene expression profile and specifically modulates oxidative stress genes (cyclooxygenase 2, glutathione peroxidases 3 and 7, and peroxiredoxin 4) and reduces the production of reactive oxygen species (ROS) in tumor cells (109). Maurer et al. (26) showed that glioma cells were not able to metabolize ketone bodies. By reducing blood glucose concentration and increasing ketonemia, KD represents a metabolic disadvantage for tumor cells. KD was used in a xenograft model as monotherapy, but without success. A therapeutic combination of KD and a glutamine antagonist, 6-diazo-5-oxo-L-norleucine (DON), was administered in two mouse models to treat orthotopic growth of advanced GBM. The effect of this pharmaconutritional approach was to reduce tumor volume, oedema, hemorrhage, inflammation and disease symptoms, improving the overall survival of the mice (110). Vallejo et al. (111) showed that acetoacetate regulates mitochondrial uncoupling protein 2 (UCP2) and induces apoptosis through caspase activation and PARP cleavage. The study also showed the synergistic effect of the low-dose (50 μM) glycolytic inhibitor 2-deoxy-D-glucose and acetoacetate, which resulted in increased cell death compared to single treatments.

**Table 1:** Preclinical studies reporting KD effects on glioma models.

Preclinical study	Results
Maurer et al. (26)	KD results in metabolic disadvantage for glioma cells, unsuccessful alone
Stafford et al. (109)	KD reduces the tumor growth rate and prolongs survival, modulates oxidative stress genes
Mukherjee et al. (110)	A combination of KD and a glutamine antagonist reduced tumor volume, oedema, hemorrhage, inflammation and disease symptoms, improving the overall survival
Vallejo et al. (111)	KD induces apoptosis through regulation of UCP2 in glioma cells
Maeyama et al. (112)	A combination of KD and bevacizumab enhanced drug effect, reduced tumor growth and increased survival

Another study demonstrating the synergistic role of KD was conducted by Maeyama et al. in 2021. They compared the effects of single treatment with bevacizumab and the combination of the drug with KD in a mouse model of glioblastoma. Diet alone showed no significant benefit, but the diet-therapy combination instead showed an enhanced effect of the drug, with reduced tumor growth and increased survival (112).

### 4.2. Clinical studies

Only two studies have been performed to evaluate the outcome of KD in pediatric patients. Nebeling et al. described the cases of two children with high-grade astrocytoma. On PET scans, an average 21.8% decrease in glucose uptake in the tumor region was observed. One patient, in particular, showed improvements in mood and developed new abilities during treatment with KD. During the 12 months of treatment, the patient showed no disease progression (19). Perez et al. (113) conducted a study of 5 pediatric patients with DIPG treated with KD for at least 3 months: they showed slightly better survival than reported in the literature (18.7 months vs. 11 months).

Recently, a study performed on 13 glioma patients evaluated a combined treatment (KD, radiotherapy and metformin): it resulted in an increased survival rate and synergistic action with radiotherapy. *In vitro*, metformin activates AMPK by inhibiting mTOR complexes, with antiproliferative activity (114). One study explored the expression of the enzymes involved in ketone body metabolism in tumor cells compared to healthy controls by immunohistochemical method. Mitochondrial ketone-limiting enzymes (OXCT1 and BDH1) were found to be low in most tumors, which could justify a better response to KD in these patients (27). Confirming this, another study using KD as monotherapy showed tumor progression in two patients with tissue expression of at least one of the two critical mitochondrial ketolytic enzymes (succinyl CoA: 3-oxoacid CoA transferase, beta-3-hydroxybutyrate dehydrogenase 1) (115). One study explored the outcome of KD in a woman with GBM. After 2 months of treatment, a 20% weight loss was observed and no tumor tissue was detected on both FDG-PET and MRI. At 10 weeks after discontinuation of KD, MRI showed tumor recurrence (116). Rieger et al. in 2014 compared the median PFS after a combined treatment with KD and bevacizumab with the PFS of standard treatment. Six of 7 (86%) patients treated with the combination protocol had a median PFS of 20.1 weeks compared to the median normal PFS of 5 weeks. A 6-month PFS of 43% was observed (117). A retrospective study investigated the effects of KD in 6 patients with GBM compared to the standard diet. KD showed a reduction in mean blood glucose in KD-treated patients in the absence of significant side effects. Four patients showed an improvement in survival (14 months) (118). A study by Strowd et al. (119) reported an increase in seizure control and survival rate in eight patients with glioma undergoing MAD. A recent study on 25 patients on an 8-week Glioma Atkins-Based Diet (GLAD) observed the accumulation of ketones in brain tissue and glioma by magnetic resonance spectroscopy. The increase in brain ketone concentration was correlated with ketone concentrations in blood and urine. Magnetic resonance spectroscopy showed an accumulation of ketones in the lesion but also in the remaining brain parenchyma (120). A study of 9 glioma patients used proton magnetic resonance imaging (1H-MRS) to assess metabolic changes during treatment with KD. Only in patients with high KD adherence were ketone bodies detected in the brain tissue, even in the area of the lesion (121). Berrington et al. stated the feasibility of 3 T magnetic resonance spectroscopy to monitor brain ketone levels in patients with high-grade gliomas. In his study, patients followed a modified Atkins diet for 8 weeks. The results showed an increase in acetone at week 8 in both the lesion and the contralateral brain and an increase in β-hydroxybutyrate at the lesion site. Acetone concentrations in the contralateral brain were strongly correlated with ketonuria and reduced fasting glucose (122).

A case report by Seyfried et al. (123) showed the effect of combined surgery and KD in treating a right frontal lobe glioma grade 4 IDH mutated. This is an example of a targeted therapy, as both KD and IDH mutation limit glioma growth acting negatively on glycolysis and glutaminolysis.

A randomized clinical trial [NCT01754350] of 50 patients evaluated the combination of radiotherapy with standard diet or caloric restriction-KD. Results published at the time showed a significantly longer PFS and OS in the restricted calories-KD group (124). Martin-McGill et al. (125) studied a larger sample of 172 glioma patients who underwent modified KD (70% kcal fat, 20 g CHO/day): KD was well tolerated, only two patients reported constipation. Another study by van der Louw et al. confirmed that the adverse effects of KD were mild and transient (126). A retrospective study of 29 patients with grade II-IV astrocytoma evaluated the use of MAD during radiotherapy and standard chemotherapy. Pre-and post-radiotherapy MRI images were evaluated for tumor pseudo progression as a marker of radiation sensitization. Ketosis was achieved in 100% of cases and no serious adverse events secondary to MAD were observed. 58% of GBM patients showed pseudo progression after MAD and radiation and temozolomide therapy (127). A particular case report described by Elsakka et al. (128) reported the effect of combined standard of care treatment along with metformin, hyperbaric treatment and KD in a patient with GBM. This innovative protocol showed a reduction in tumor invasion and symptoms of GBM, indicating that the successful outcome could be attributed to, among other things, KD. As evidence of the feasibility of KD, the KEATING study was performed to investigate the use of KD as an adjuvant therapy for 12 patients with GBM. A modified ketogenic diet (MKD) or a ketogenic diet with medium-chain triglycerides (MCTKD) were used. Recruitment and maintenance rates were low: only four patients completed the three-month diet (MCTKD *n* = 3; MKD *n* = 1). The outcome of these patients needs to be further evaluated (129).

## 5. Conclusion

The incidence of brain tumors in children is approximately 5 cases per 100,000 population, 75% of which are classified as gliomas. Genetic alterations have been extensively studied in recent years and are associated with the prognosis and clinical course of glioma. The use of alternative and complementary therapies in pediatric cancer patients, as the pre-clinical data has encouraging, seem to be a frequent choice by the. In recent years, there has been growing scientific interest in the use of ketogenic diets (KDs) as a complementary approach to standard cancer therapy, particularly in central nervous system tumors. The biochemistry of this diet is quite well known. The hepatocytes in the liver convert fats from the diet into fatty acids and ketone bodies, allowing the latter to be utilized as alternative sources of energy. *In vitro* studies have shown that glioma cells are unable to compensate for glucose restriction by metabolizing ketone bodies, suggesting a potential disadvantage of tumor cells compared to normal cells. Dietary compounds are important epigenetic modulators and increasing evidence suggests that epigenetic changes are also associated with the ketogenic diet. KD may be able to enhance the transcription of genes, both directly and indirectly, through epigenetic marks such as DNA methylation, modification of histone conformation by acetylation, methylation, phosphorylation, ubiquitylation and lysine beta-hydroxybutyrylation. Increased production of reactive oxygen species (ROS) and oxidative stress at baseline is also a consistent phenotype in tumors of all tissue types. Many studies have shown that the ketogenic diet elevates oxidative stress in tumor cells, while simultaneously being neuroprotective in reducing the production of reactive oxygen species. For the host is crucial counter foreign pathogens or tissue damage through the inflammasome activation, however excessive and aberrant inflammasome activation can otherwise cause uncontrolled tissue responses that contribute to various diseases, including cancer. The identification of β-hydroxybutyrate as an NLRP3 inhibitor and the well known anti-inflammatory properties of this diet appear to be a consistent perspective themes of investigation. A substantial number of preclinical and clinical studies have been performed to evaluate the effect of KD on low-grade and high-grade glioma. In pediatric age, only two descriptive studies have been conducted, but RCT and large sample studies are lacking.

It is useful to emphasize that defining childhood cancer as an essentially epigenetic and dysontogenetic disease means paying more attention to procarcinogenic environmental factors capable of interfering early on in the epigenetic programming of cells, tissues and organs by inducing reactive/dis-reactive changes in proliferative and differentiative programs (*fetal programming*) (130). Moreover, accepting such a perspective means placing greater value on nutritional factors in both carcinogenic patterns and therapeutic approaches.

## Author contributions

NC and EB: study conception and design. RR and FR: data collection, analysis and interpretation of results, and draft manuscript. MA: data collection, analysis and interpretation of results. All authors reviewed the results and approved the final version of the manuscript.

## Conflict of interest

The authors declare that the research was conducted in the absence of any commercial or financial relationships that could be construed as a potential conflict of interest.

## Publisher’s note

All claims expressed in this article are solely those of the authors and do not necessarily represent those of their affiliated organizations, or those of the publisher, the editors and the reviewers. Any product that may be evaluated in this article, or claim that may be made by its manufacturer, is not guaranteed or endorsed by the publisher.
